# Associations of NETs with inflammatory risk and clinical predictive value in large artery atherosclerosis stroke: a prospective cohort study

**DOI:** 10.3389/fimmu.2024.1488317

**Published:** 2024-12-16

**Authors:** Jiang Li, Lei Liu, Ruxu Zhang, Liqun Pan, Juanying Tan, Mingxin Ou, Xiuju Luo, Jun Peng, Zhongyang Hu

**Affiliations:** ^1^ Health Management Medical Center, The Third Xiangya Hospital, Central South University, Changsha, Hunan, China; ^2^ Department of Neurology, The Third Xiangya Hospital, Central South University, Changsha, Hunan, China; ^3^ Department of Clinical Laboratory, The Third Xiangya Hospital, Central South University, Changsha, Hunan, China; ^4^ Department of Pharmacology, Xiangya School of Pharmaceutical Sciences, Central South University, Changsha, China

**Keywords:** large artery atherosclerosis stroke, neutrophil extracellular traps, inflammation, prognosis, major vascular events

## Abstract

**Background and objective:**

Neutrophil extracellular traps (NETs) with inflammatory risk are important contributors to cardiovascular disease, but no definitive information is available in large artery atherosclerotic (LAA) stroke. This study aims to investigate the association between NETs with related inflammatory biomarkers and prognosis of LAA stroke in the Chinese population.

**Methods:**

A prospective study involving 145 LAA stroke cases and 121 healthy controls was conducted. Serum levels of MPO-DNA, PAD4, HMGB1, C1q, AIM2, ASC, Caspase-1, IL-1β, IL-6, and IL-8 were determined in all participants. The biomarkers were detected at three time points after stroke onset (24 hours: T1, 48 hours: T2, 7 days: T3) for LAA stroke patients and once for controls. Patients were followed up for 2 years after the ischemic event.

**Results:**

The serum MPO-DNA, PAD4, C1q, IL-1β, IL-6 and IL-8 reach their peak at 24 hours after stroke onset and show a decreasing trend during acute phase. MPO-DNA, AIM2 and IL-1β at baseline were associated with poor outcome at 3 months, further GMDR analysis revealed that the combination of MPO-DNA, AIM2 and IL-1β exert a synergistic effect on the prognosis of LAA stroke (OR: 8.75 95%CI (2.10-32.42)). For time-to-event analysis, MPO-DNA, Caspase-1 and IL-1β at baseline were predictors of MVEs after stroke (HR:4.04 (95%CI 1.28-12.70), 2.33 (95%CI 1.06-5.12) and 4.09 (95%CI 1.39-11.99), respectively).

**Conclusions:**

NETs and related inflammatory biomarkers at baseline predicted outcome at 3 months and late major vascular events following LAA stroke, supporting a rationale of randomized trials for targeted therapy directed at high-risk patients with elevated baseline NETs and related inflammatory biomarkers.

## 1 Introduction

Ischemic stroke is the major cause of death and disability worldwide (1). The prevalence and the burden of ischemic stroke is marked increased in China (2). Despite the widespread use of contemporary secondary prevention therapy, there is an inevitable residual vascular risk in ischemic stroke survivors. In Chinese population, the 5-years cumulative rate of recurrent ischemic stroke and the rate of major vascular events (MVEs) at 5 years were 41% and 45%, respectively (3). Notably, patients with large artery atherosclerosis (LAA) stroke have a higher residual risk of stroke recurrence than other subtypes of ischemic stroke (4, 5). It is indicated that the residual risk of recurrent stroke may partial attributed to inflammation (6–8). There is an urgent requirement for novel prognostic biomarker to predict the residual risk following LAA stroke, which will pave the way for precise treat and prevention of LAA stroke in the future. Therefore, we aimed to assess the value of inflammatory markers in predicting this residual vascular risk following LAA stroke.

In recent decades, a plethora of studies have provided new data highlighting the role of inflammation in the pathogenesis of LAA stroke, such as atherogenesis and atherothrombosis (9–12). Neutrophils are believed to be the predominant leukocyte population in human blood and the first cells recruited to an inflammatory site, such as ischemic brain tissue (13). Under the influence of inflammatory cytokines, neutrophils further form neutrophil extracellular traps (NETs), which are composed of extracellular DNA, histones, and granular and cytoplasmic proteins, such as myeloperoxidase (MPO), neutrophil elastase (NE), Peptidyl arginine deiminase 4 (PAD4), etc. The mechanistic understanding of NET formation in neutrophils is uncovered by recent studies (14, 15). Rupture of the nuclear envelope, chromatin decondensation, loading of the chromatin with granular and cytoplasmic proteins, and plasma membrane breakdown are key cellular events for the release of chromatin during NET formation (16–19). The MPO-DNA complex is considered to be one of the more specific markers currently available for NETs formation assessment (20). Basic research revealed that NETs may further exacerbated ischemic brain injury (21). However, the association between circulating NETs concentration and the prognosis of ischemic stroke patients is controversial, making the role of NETs in cerebrovascular disease an intriguing topic (22, 23).

Recent studies indicate that the prognosis of ischemic stroke is linked to the global inflammatory response in the brain (24, 25). Interleukin-1β (IL-1β) is not only the core of inflammatory response in the brain after ischemic stroke, but also as a key driver of innate immune memory, which result in chronic post-stroke comorbidities (16, 20–22). Understanding the mechanisms regulating production of IL-1β during ischemic brain injury may lead to the identification of new therapeutic targets. The activity of interleukin-1 is regulated by multi-molecular protein complexes called inflammasome, such as AIM2 (absent in melanoma 2) inflammasomes and NLRP3 (NLR family, pyrin domain containing 3) inflammasome, etc. (29). Using a rodent model of stroke, Denes et al. (30) revealed that AIM2 inflammasomes contribute to brain injury, and that the NLRP3 inflammasome is not involved in this process. Further researches indicated inhibiting the NETs/AIM2 axis may be a potential strategy to reduce inflammatory damage to target organs (31, 32), additional evidence confirmed this double-strand DNA/AIM2 axis may regulate atherosclerotic plaque vulnerability (33). Enhanced plaque inflammation results in plaque destabilization and atherothrombosis. Besides the pro-inflammatory property, pro-thrombotic activity of extracellular DNA can be driven by aggregated NETs, which provide a scaffold for thrombus formation and are able to occlude vessels (15, 34, 35). Furthermore, NETs-associated occlusions have been reported for coronary vessels in acute myocardial infarction and atherosclerosis (36, 37) and for cerebral vessels in ischemic stroke (38). Therefore, investigation the prognostic value of markers in the NETs/AIM2 inflammasome axis, such as MPO-DNA, PAD4, HMGB1, C1q, AIM2, ASC, Caspase-1, IL-1β, IL-6 and IL-8, in LAA stroke patients is a meaningful endeavor.

In this prospective study, we aim to decipher the dynamic change pattern of circulating biomarker in NETs/AIM2 inflammasome pathway at acute stage of LAA stroke. We further explored the associations between these indicators and prognosis of LAA stroke, which might promote the accurate prevention and treatment of LAA stroke in the future.

## 2 Methods

### 2.1 Study design and participants

Between December 2020 and March 2023, patients aged ≥18 years with acute LAA stroke diagnosed by Chinese Guidelines for the Diagnosis and Treatment of Acute Ischemic Stroke (2018) in the Third Xiangya Hospital of Central South University were enrolled (39). We enrolled LAA stroke patients within 24 hours of symptom onset. Exclusion criteria included: (1) combined intracranial hemorrhagic diseases; (2) transient ischemic attack; (3) concomitant acute myocardial infarction; (4) active liver disease or hepatic dysfunction, defined as aspartate aminotransferase (AST), or alanine aminotransferase (ALT) ≥2×the upper limit of normal (ULN); severe renal dysfunction, glomerular filtration rate (GFR) <30ml/min/1.73m^2^; severe coagulation disorders and hematological diseases; malignant tumors; autoimmune diseases; heart failure and respiratory failure. (5) childbearing and pregnancy women; (6) merging diseases such as Parkinson’s disease, Alzheimer’s disease, Amyotrophic lateral sclerosis, Friedrich’s ataxia, etc.(7) recent history of trauma and surgery; (8) incomplete clinical data. All eligible patients were enrolled consecutively in this study to eliminate selection bias.

**Figure 1:**
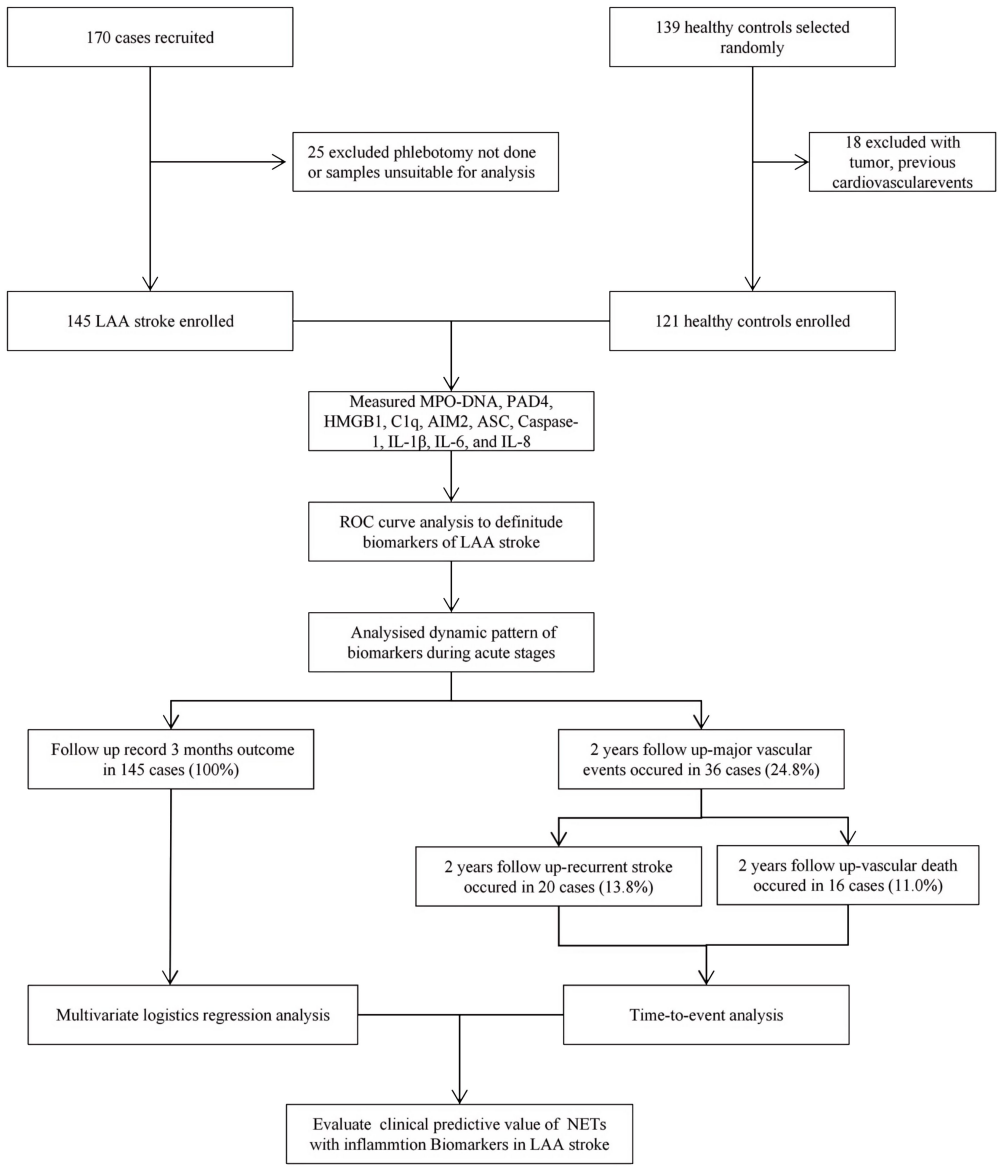
The flow chart of patient enrollment for this study. LAA stroke, large artery atherosclerotic (LAA) stroke; IL, interleukin; MPO, myeloperoxidase; PAD4, Peptidyl arginine deiminase-4; HMGB1, High mobility group box-1 protein; C1q, Complement component 1q; AIM2, absent in melanoma 2; ASC, Apoptosis associated speck like protein containing CARD.

Clinical follow-up was performed two years after stroke onset in 145 of the 170 initial patients (as shown in **Figure 1**). This was conducted by reviewing the electronic records of our patients in clinics or by telephonic contact. We consecutively enrolled 121 healthy individuals with no history of stroke or other severe diseases, who underwent health check-ups during the same period as the enrollment of LAA stroke patients. This prospective cohort study was approved by the Ethics Committee of the Third Xiangya Hospital of Central South University (No.: 22125) and all participants have provided written informed consent.

### 2.2 Clinical variables

The clinical variables collected for this study included age, sex, history of hypertension and diabetes mellitus type 2 (T2DM), history of smoking, body mass index (BMI), high density lipoprotein cholesterol (HDL-C), low density lipoprotein cholesterol (LDL-C), triglyceride (TG), total cholesterol (TC), and homocysteine (Hcy). Peripheral venous blood was obtained from LAA stroke patients at 24 hours after the onset of the acute event (T1) and from the healthy subjects after a night fast. In LAA stroke patient group, the next two time points of collecting peripheral venous blood were T2 (48 hours after stroke onset) and T3 (7 days after stroke onset). HDL-C, LDL-C, TG, TC, and Hcy were detected in all patients only once at 24 hours after stroke onset by standard methods in the Clinical Laboratory of the Third Xiangya Hospital of Central South University.

### 2.3 Laboratory methods

Venous blood samples were collected at three time points (T1, T2 and T3) for LAA stroke patients and once for controls. Serum was centrifuged within an hour of sampling (2500 rpm for 10 min), and stored at −80°C pending analysis. The serum samples Enzyme Linked Immunosorbent Assay (ELISA) was applied to measure the levels of MPO-DNA, PAD4, HMGB1, C1q, AIM2, ASC, Caspase-1, IL-1β, IL-6 and IL-8 with ELISA kits (Quanzhou Ruixin Biotechnology Company, Quanzhou, China) according to the manufacturer’s instructions.

### 2.4 Stroke related scale and follow-up

The severity of LAA Stroke patients was assessed by the National Institutes of Health Stroke Scale (NIHSS) at admission. Patients were followed-up for 2 years after the ischemic event. During the 3 months follow-up, the Modified Rankin Scale (mRS) was used to evaluate the prognosis condition. These scales were evaluated by trained and qualified neurologists. We categorized them into two groups based on their mRS scores: an mRS score of ≤2 was defined as the good outcome group, while an mRS score of >2 was defined as the poor outcome group. The 2-year follow-up was conducted by telephone calls or in-person interview during outpatient visits. In time-to-event analysis we recorded major vascular events as clinical endpoints. Major vascular events were defined as recurrent ischemic stroke, myocardial infarction or vascular death, whichever was reported first.

### 2.5 Statistical analysis

SPSS 26.0, GraphPad Prism 9.5 and GMDR 0.9 were used for statistical analysis and data visualization. First, Kolmogorov-Smirnov test was used to determine whether the measurement data obey the standard normal distribution. The means ± SEM and the median (interquartile distance) was used to represent the measurement data, and counting data were expressed as percentages (%). We compared continuous variables using t-test or Mann-Whitney test as appropriate. Categorical variables are reported as count (percentage). We compared categorical variables by χ2 test or Fisher exact test. After the homogeneity test of variance, the mean of multiple groups was compared using single factor repeated measure analysis of variance (ANOVA). For correlation analysis, Spearman rank correlation was applied to indicators that were not normally distributed. Receiver Operator Characteristic (ROC) was used to analyze the prognostic value of all relevant indicators in LAA stroke patients. Multivariate logistic regression analyses were used to assess the independent association between inflammatory biomarkers and clinical outcome. To further elucidated the potential synergistic effect, we employed the generalized multifactor dimensionality reduction (GMDR) method to detect interactions between inflammatory factors. Multivariate cox regression was used to analyze the risk factors of clinical endpoints, which estimates the hazard ratio (HR) of a given endpoint associated with a specific risk factor. A two- tailed *P* value less than 0.05 was considered statistically significant.

## 3 Results

### 3.1 Baseline characteristics

**Figure 2:**
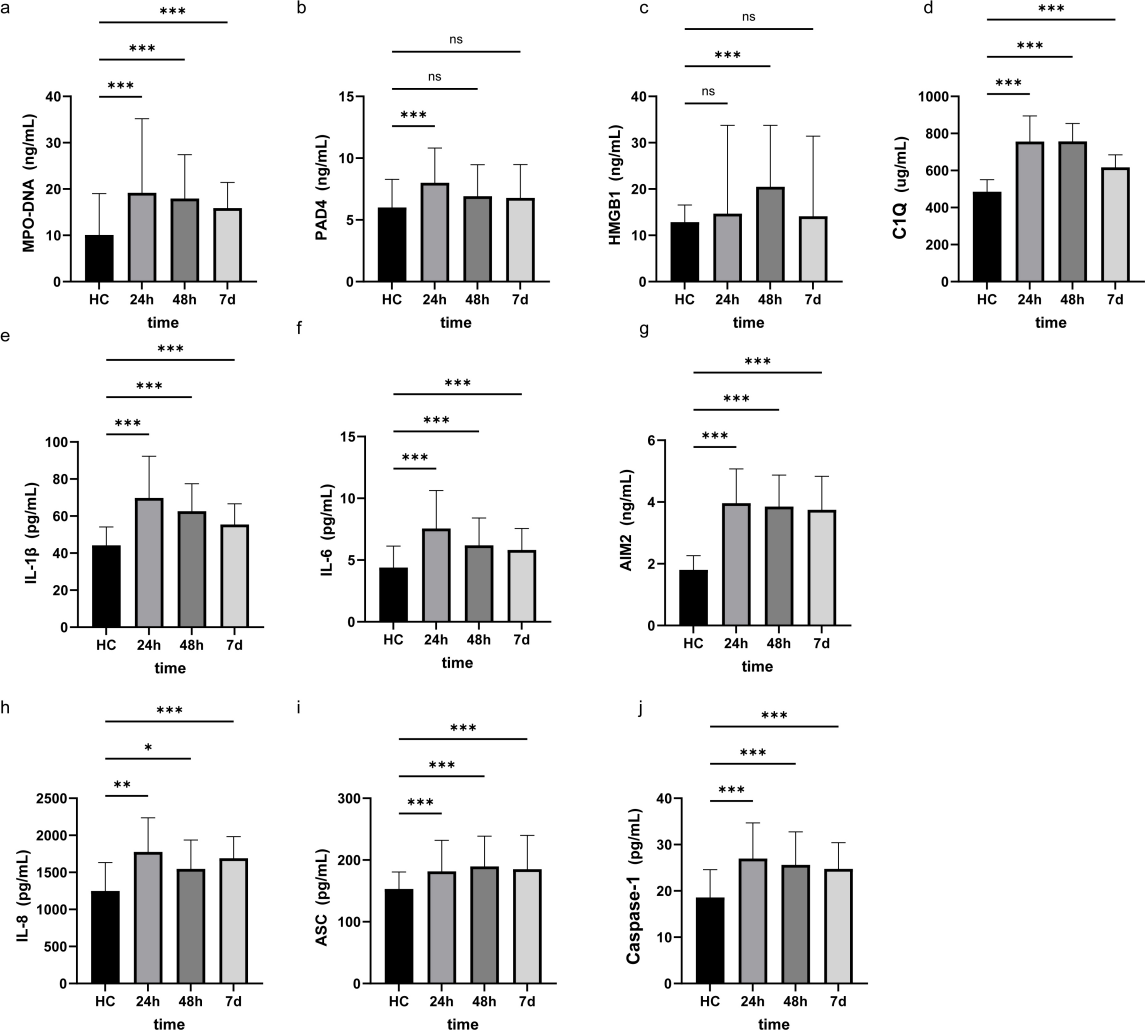
Research factor level at three different time points in LAA stroke and Healthy controls. The results were obtained using Mann-Whitney U. HC, Healthy controls *: *p*<0.05, **: *p*<0.01, ***: *p*<0.001, ns: no significance. 24h, 24 hours after LAA stroke onset; 48h, 48 hours after LAA stroke onset; 7d, 7 days after LAA stroke onset; MPO, myeloperoxidase; PAD4, Peptidyl arginine deiminase-4; HMGB1, High mobility group box-1 protein; C1q, Complement component 1q; AIM2, absent in melanoma 2; IL-1β, interleukin-1β; IL-6, interleukin-6; IL-8, interleukin-8; ASC, Apoptosis associated speck like protein containing CARD.

**Figure 3:**
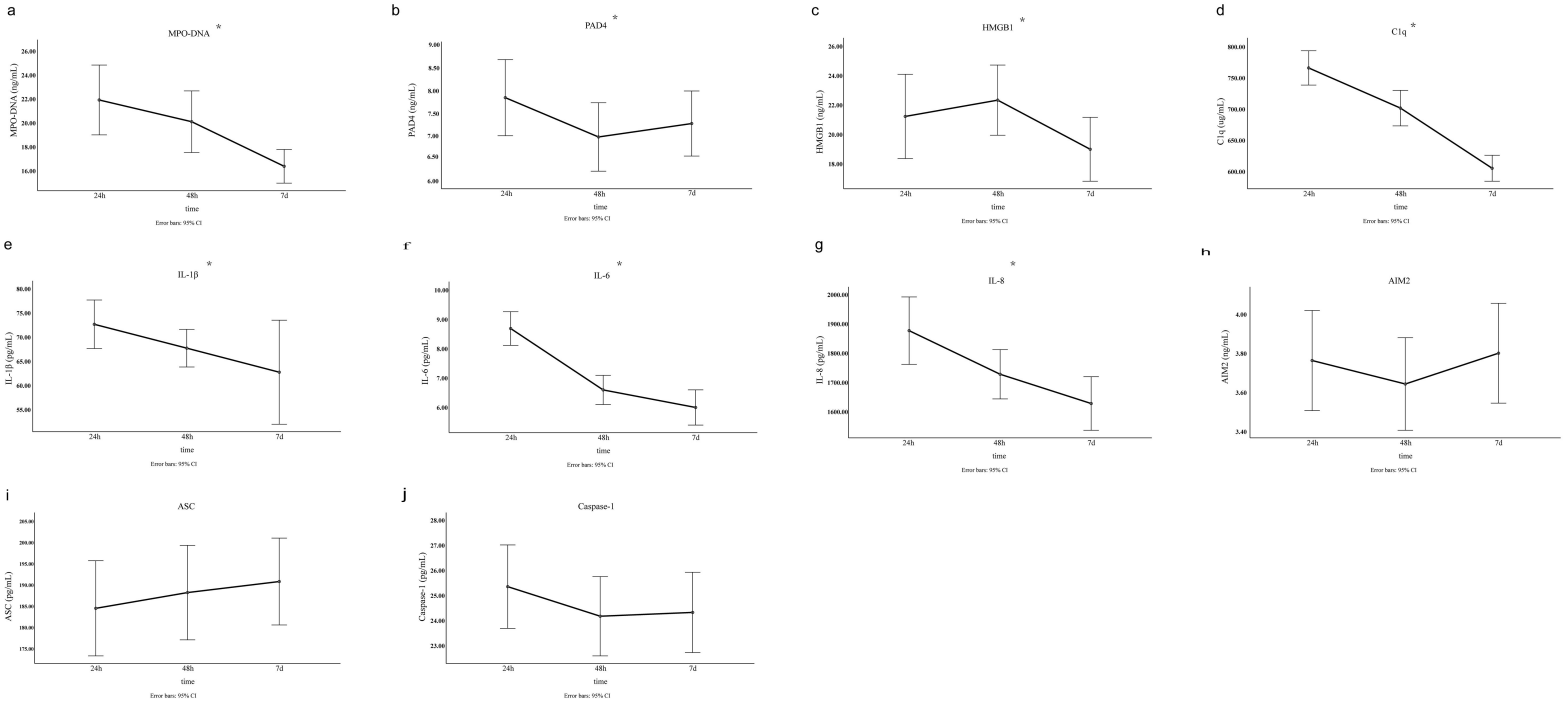
The level of NETs and inflammatory biomarkers at three different time points in the acute phase of LAA stroke. The results were obtained using single factor repeated measure analysis (ANOVA). 24h, 24 hours after LAA stroke onset; 48h, 48 hours after LAA stroke onset; 7d, 7 days after LAA stroke onset; MPO, myeloperoxidase; PAD4, Peptidyl arginine deiminase-4; HMGB1, High mobility group box-1 protein; C1q, Complement component 1q; AIM2, absent in melanoma 2; IL-1β, interleukin-1β; IL-6, interleukin-6; IL-8, interleukin-8; ASC, Apoptosis associated speck like protein containing CARD. *: *p*<0.05.

Comparison of clinical baseline characteristics between LAA stroke group and healthy control group is shown in **Supplementary Table 1**. The age and sex between the two groups were comparable (*P*>0.05). Of the 145 LAA stroke patients, 98 were males (67.6%) and 47 were females (32.4%), at average age of 65.0 years old. Among these patients, 46 (31.7%) had a history of smoking, 109 (75.2%) had hypertension, and 40 (27.6%) had diabetes mellitus type 2 (T2DM). Among patients with LAA stroke, the prevalence of smoking history, hypertension, and T2DM was significantly higher than that observed in the healthy controls (all *P*<0.05). The TG level of LAA Stroke patients was significantly higher than in healthy controls (P = 0.025). Similarly, The TC level of LAA Stroke patients was significantly higher than in healthy controls (P = 0.031). There was no significant difference in the LDL-C and HDL-C between the groups. Comparison of the serum biomarkers level between LAA stroke group and healthy control group is shown in **Figure 2**. The levels of MPO-DNA, C1q, AIM2, ASC, Caspase-1, IL-1β, IL-6 and IL-8 were significantly higher in LAA stroke group than in control group at 3 time points (all *P*<0.05). The levels of MPO-DNA, PAD4, C1q, IL-1β, IL-6, IL-8 and HMGB1 change significantly over time during the acute phase of stroke (all *P* < 0.05) (as shown in **Figure 3**). For detail, MPO-DNA, PAD4, C1q, IL-1β, IL-6 and IL-8 reached their peak at 24 hours after stroke onset and show a decreasing trend throughout the acute phase of stroke, while HMGB1 peaked at 48 hours after stroke onset. The levels of AIM2, ASC and Caspase-1 do not change significantly over time during the observation timeframe (all *P*>0.05) (as shown in **Figure 3**). Correlation analysis between NETs and inflammatory biomarkers is shown in **Supplementary Figure 1**. Especially, MPO-DNA was positively correlated with PAD4, HMGB1, C1q, AIM2 (*P*=0.002, r=0.260; *P*=0.002, r=0.251; *P*=0.001, r=0.322; *P*=0.019, r=0.195, respectively). Furthermore, HMGB1 at T1 was correlated with NIHSS scores at hospital admission (P=0.009, r=0.217, as shown in **Supplementary Table 2**). At T2 and T3, correlation analysis revealed no statistically significant associations between any of the biomarkers and NIHSS scores at hospital admission (*P* > 0.05) (**Supplementary Table 2**).

### 3.2 Multivariate logistic regression analysis of predictors for three months prognosis in LAA stroke patients

**Figure 4:**
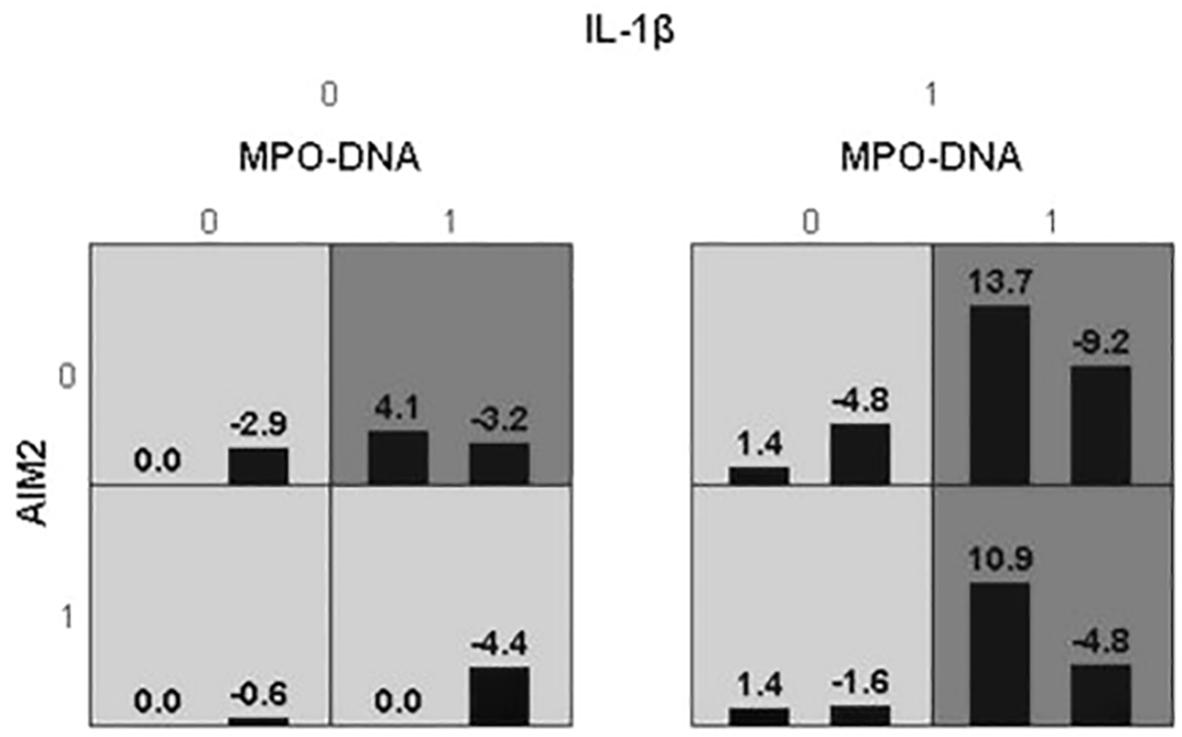
The best model for ischemic stroke identified by GMDR. The best model, which is composed of MPO-DNA, AIM2, IL-1β may statistically increase the risk of ischemic stroke. In GMDR analysis, the three-loci combinations were classified into high- or low-risk groups. The background shading within each cell indicates the risk to ischemic stroke of each given combination. High-risk cells are indicated by dark shading, low-risk cells by light shading, and empty cells by white color. Based on the chi-squared test, the OR value of the high-risk combinations of the synergistic effects model indicated an increased risk of poor prognosis of LAA stroke (*P*=0.0107, OR: 8.75 95%CI (2.10-32.42)). Cl, confidence interval; OR, odds ratio; GMDR, generalized multifactor dimensionality reduction.

Receiver Operator Characteristic (ROC) was used to analyze the prognostic value of all relevant indicators in LAA stroke patients (as shown **Supplementary Table 3**). According to the mRS Scores at 3 months after stroke onset, multivariate logistic regression analysis found that high levels of MPO-DNA, AIM2, and IL-1β at 24 hours after stroke onset were associated with poor prognosis in LAA stroke patients (*P*=0.007, OR (95%CI): 4.88 (1.53, 15.5), *P*=0.012, OR (95%CI): 2.78 (1.25, 6.17), and *P*=0.017, OR (95%CI): 3.44 (1.24, 9.49), respectively), and coexisting T2DM were associated with poor prognosis (*P*<0.05) (as shown in **Table 1**). To further elucidated the potential synergistic effect, we employed the GMDR method to detect interactions between inflammatory factors. The model composed of MPO-DNA, AIM2 and IL-1β had the highest level of testing balance accuracy (0.585), the cross-validation consistency of 7/10, and scored 9 for the sign test at the 0.0107 level, as shown in **Table 2**. Furthermore, the significant interactions between the above models were confirmed by a permutation test implemented in the GMDR software (*P*=0.028). Further analysis revealed that combination of MPO-DNA, AIM2 and IL-1β exert a synergistic effect on the prognosis of LAA stroke [*P*=0.001, OR: 8.75 95%CI (2.10-32.42)] (**Figure 4**). At T2 and T3, multivariate logistic regression analysis demonstrated that the associations between NETs and inflammatory biomarkers and stroke prognosis were not statically significant(*P*>0.05) (**Supplementary Tables 4**, **5**).

**Table 1:** Logistic regression analysis of inflammatory biomarkers at 24 hours after LAA stroke onset and poor clinical outcomes.

Variables	Univariate			Multivariate		
	OR	95%CI	*P*	OR	95%CI	*P*
Age(≥60)	2.65	1.18-5.64	0.018	–	–	–
Sex(female)	2.35	1.13-4.90	0.022	–	–	–
Hypertension T2DM	1.07 3.04	0.47-2.42 1.41-6.51	0.862 0.004	- 2.68	- 1.16-6.21	- 0.021
Smoke	0.67	0.31-1.46	0.322	–	–	–
BMI (≥28kg/m^2^)	1.22	0.38-3.86	0.736	–	–	–
LDL-C (≥3.4mmol/L)	1.35	0.55-3.21	0.512	–	–	–
HDL-C(≤1.16mmol/L)	0.93	0.45-1.93	0.851	–	–	–
Triglycerides (≥1.7mmol/L)	0.86	0.42-1.77	0.693	–	–	–
Cholesterol (≥5.2mmol/L)	1.11	0.51-2.38	0.790	–	–	–
Hcy (≥15mmol/L)	1.53	0.69- 3.36	0.288	–	–	–
MPO-DNA (≥11.5ng/mL)	4.78	1.57-14.52	0.006	4.88	1.53-15.5	0.007
PAD4 (≥6.78ng/mL)	1.32	0.64-2.74	0.447	–	–	–
HMGB1 (≥21.9ng/mL)	1.13	0.55-2.30	0.738	–	–	–
C1q (≥604.87ug/mL)	0.54	0.23-1.27	0.162	–	–	–
AIM2 (≥2.6ng/mL)	2.54	1.23-5.25	0.012	2.78	1.25-6.17	0.012
ASC (≥202.52pg/mL)	1.12	0.54-2.31	0.748	–	–	–
Caspase-1 (≥28.92pg/mL)	1.08	0.52-2.20	0.815	–	–	–
IL-1β (≥60.91pg/mL)	3.64	1.40-9.44	0.008	3.44	1.24-9.49	0.017
IL-6 (≥7.14pg/mL)	0.80	0.39-1.61	0.533	–	–	–
IL-8 (≥1736.92pg/mL)	0.81	0.37-1.76	0.601	–	–	–

OR, Odds Ratio; CI, Confidence Interval.

**Table 2:** GMDR results of multi-factors interaction with ischemic stroke.

Factors	Model	Training Balance Accuracy	Testing Balance Accuracy	CV Consistency	Sign Test(p)
1	MPO-DNA	0.6252	0.5103	5/10	8(0.0547)
2	MPO-DNA IL-1β	0.6768	0.577	6/10	9(0.0107)
3	MPO-DNA AIM2 IL-1β	0.6933	0.585	7/10	9(0.0107)
4 5	MPO-DNA AIM2 ASC IL-1β MPO-DNA PAD4 AIM2 Caspase-1 IL-1β	0.7308 0.7746	0.5572 0.5553	5/10 2/10	5(0.6230) 6(0.3770)

### 3.3 Multivariate Cox regression analysis of predictors for clinical endpoints in LAA stroke patients

**Figure 5:**
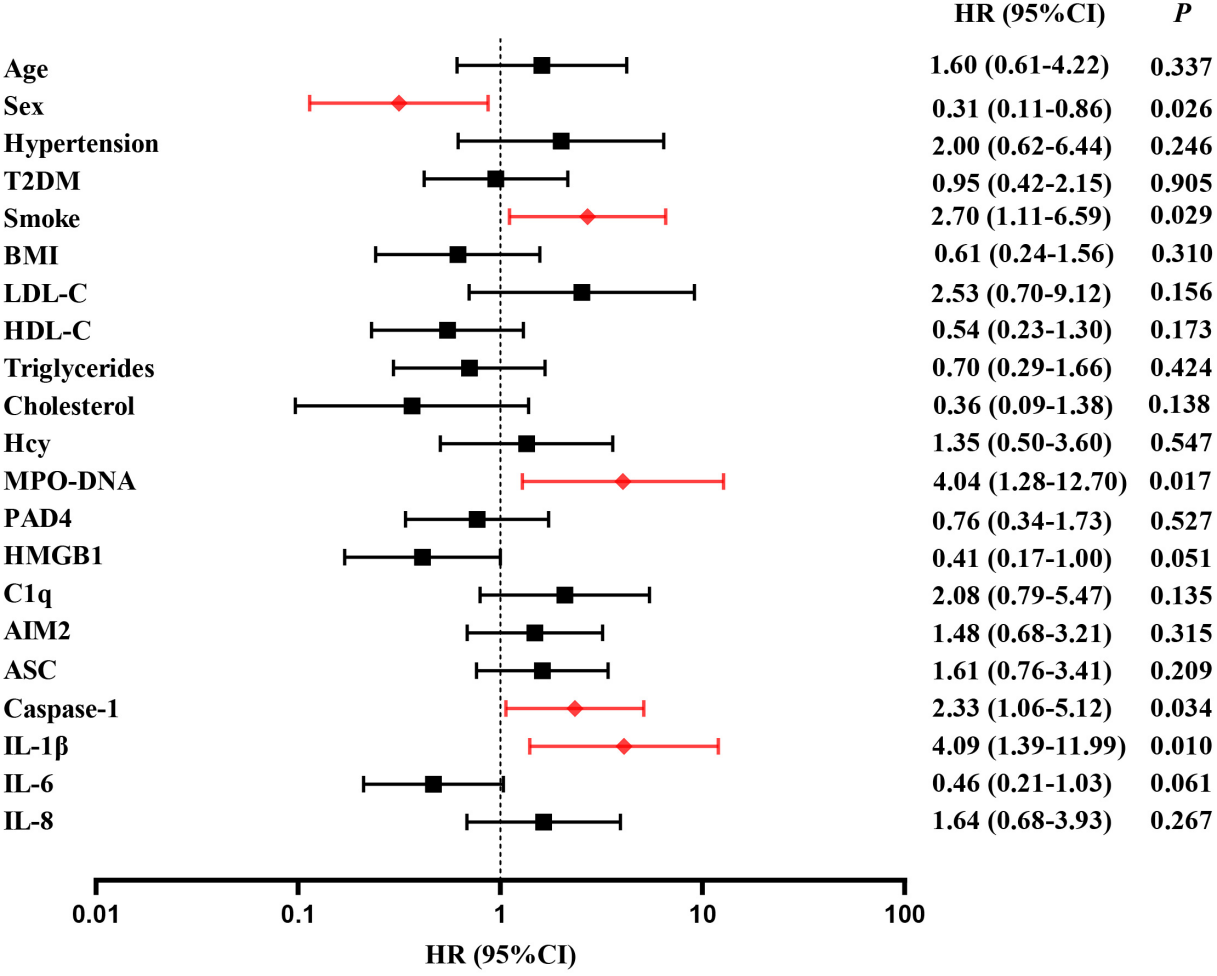
Time to event analysis of risk factors for MVEs in LAA stroke. HR, hazard ratio.

**Figure 6:**
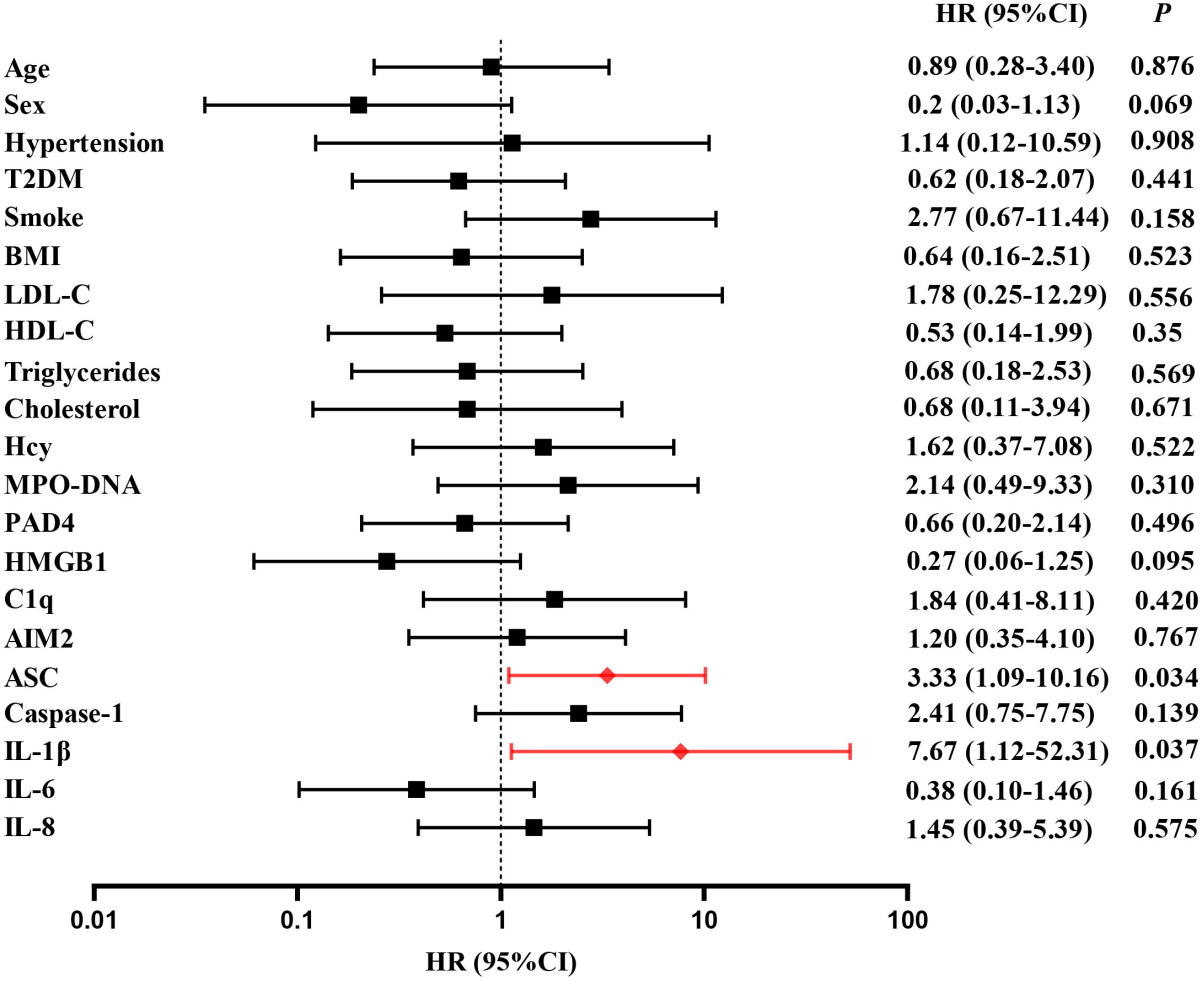
Time to event analysis of risk factors for recurrence in LAA stroke. HR, hazard ratio.

A follow-up was conducted to document major vascular events occurring 2 years post-stroke onset in 145 patients. 36 patients (24.8%) experienced major vascular events, including 20 patients with recurrent ischemic stroke (13.8%) and 16 patients with vascular death (11.0%). For detail, 10 patients died from ischemic stroke and 6 patients died from acute myocardial infarction. Multivariate cox regression was used to analyze the risk factors of clinical endpoints, which estimates the hazard ratio (HR) of a given endpoint associated with a specific risk factor. Multivariate cox regression demonstrated high level of MPO-DNA, Caspase-1 and IL-1β at baseline were risk factors of MVEs after LAA stroke(*P*=0.017, HR (95%CI): 4.04 (1.2, 12.70), *P*=0.034, HR (95%CI): 2.33 (1.06, 5.12), and *P*=0.01, HR (95%CI): 4.09(1.39, 11.99), respectively)). Additionally, the analysis showed female and smoke predictors of MVEs (*P*=0.026, HR (95%CI): 0.31 (0.11, 0.86), *P*=0.029, HR (95%CI): 2.70 (1.11, 6.59), respectively) (**Figure 5**). Furthermore, high level of ASC, IL-1β at baseline were risk factors of recurrent stroke (*P*=0.034, HR (95%CI): 3.33 (1.096,10.161), and *P*=0.037, HR (95%CI): 7.67 (1.126,52.31), respectively) (**Figure 6**).

## 4 Discussion

Recent basic studies have identified that neutrophil extracellular traps (NETs) as a potential inflammatory biomarker in ischemic stroke (27, 38, 40–42). Therefore, exploring the role of NETs in the prognosis of cerebrovascular disease patients holds significant promise for advancing our understanding of these conditions. However, the association between NETs and ischemic stroke prognosis remains controversial, making their role in ischemic stroke an intriguing topic, which warrant further investigation (22, 23). Our results revealed, in multivariate logistic analysis, baseline levels of MPO-DNA, AIM2, and IL-1β were predictors of poor outcome at three-months in LAA stroke patients. To further elucidated the potential synergistic effect, we employed the GMDR method to detect interactions between inflammatory factors. The GMDR analysis revealed that the combination of MPO-DNA, AIM2, and IL-1β exert a synergistic effect on the prognosis of LAA stroke, which indicated NETs/AIM2/IL-1β axis may involve in inflammatory damage after ischemic stroke. It is noteworthy that similar findings have been observed in clinical cardiovascular study, which revealed an association between high levels of NETs and inflammatory responses in ST-segment elevation myocardial infarction (STEMI) (43). Additionally, circulating NETs have significant predictive value for left ventricular remodeling in myocardial infarction patients (40, 43). The above evidence indicated that NETs could serve as prognostic biomarkers for clinical outcome after both cerebrovascular and cardiovascular disease.

Although few study focus on the dynamic changes of NETs and related inflammatory biomarkers in human peripheral blood during the acute phase of ischemic stroke, De Wilde et al. (41) used a middle cerebral artery occlusion (MCAO) rat model to described the dynamic change pattern of NETs, which could be detected in the ipsilateral brain hemisphere of MCAO rats 6 hours after stroke, peaked at 24 hours, and decreased again 48 hours postischemia. Based on above evidence, we hypothesized that circulating NETs levels would peak approximately 24 hours post-onset of LAA stroke. To investigate this, we examined the dynamic fluctuations in NETs and related inflammatory biomarkers in peripheral blood samples collected from 24 hours to 7 days following LAA stroke onset. For the first time, our findings revealed that circulating NETs, PAD4, C1q, IL-1β, IL-6 and IL-8 levels peaked at 24 hours after stroke onset, followed by a gradual decline. These results suggest that NETs formation and inflammation may be initiated in the early stage of LAA stroke, underscoring a potentially optimal timing to target NETs related inflammation in LAA stroke in future research. However, certain biomarkers peaked at different time points, and the dynamic patterns of some biomarkers remain unclear within the observed time frame in our study.

In future studies, we propose extending the observation timeframe to encompass both the initial 24 hours and the period beyond 7 days, to characterize more information about the dynamic changes of predictive biomarkers and pave the way for precise treatment and prevention of LAA stroke in the future.

Our study provides novel clinical evidence supporting the role of the NETs/AIM2 inflammasome/IL-1β axis in the prognosis of LAA stroke. Comparably, basic study indicated a significant correlation between NETs levels and IL-1β levels, inhibiting the NETs/AIM2 axis may be a potential strategy to reduce inflammatory damage to target organs (25, 26). AIM2 is regarded as a novel receptor for cytoplasmic DNA, such as NET-DNA (32). After AIM2 inflammasome activation, AIM2 forms an inflammasome with its ligand and ASC to activate caspase-1, which controls the catalytic cleavage of the cytokine IL-1β, indicating that NETs/AIM2 inflammasome/IL-1β axis is involved in sterile inflammation related to tissue damage (32, 44). Recent preclinical evidence suggested inhibition of inflammation markers in this axis, such as NETs and AIM2, could alleviate neuroinflammatory responses and improves cerebral blood flow perfusion in ischemic brain tissues in MCAO rat (21, 24), which further supported our findings. Our results suggest that future randomized controlled trials are warranted to evaluate the safety and efficacy of targeting NETs, AIM2, or IL-1β for improved stroke outcomes.

How to predict the risk of recurrent stroke and MVEs following ischemic stroke using biomarkers is an unsolved issue. The Stroke Prevention by Aggressive Reduction in Cholesterol Levels (SPARCL) studies (6, 7) have demonstrated that despite optimal lipid-lowering therapy, a subset of patients continue to experience recurrent stroke. Series of studies have provided evidence suggesting that inflammatory biomarkers may serve as potential predictors of residual vascular risk following ischemic stroke (7, 8, 45). The underlying mechanisms remain unclear, but recent basic studies have provided some clues. Firstly, IL-1β is not only the core of inflammatory response in the brain after ischemic stroke, but also as a potential driver of innate immune memory (28), which may result in chronic post-stroke comorbidities, such as major vascular events. Furthermore, recent experimental research has revealed that NETs are associated with amplified immune cell recruitment in atherosclerotic plaques and the formation of vulnerable plaques, thereby increasing the risk of vascular events (46–49). While NETs have been identified as biomarkers of acute stroke, their role in post-stroke MVEs remains unclear (22, 23). Intriguingly, our study revealed a novel association between high levels of circulating NETs, Caspase-1, and IL-1β at baseline and the occurrence of MVEs in our cohort, suggesting that NETs may contribute to the residual vascular risk following LAA stroke. Additionally, our study also revealed that elevated ASC and IL-1β levels were associated with an increased risk of recurrent stroke. We proposed that the NETs, ASC, Caspase-1, IL-1β pathway is an important regulator of post-stroke inflammation which might exacerbate atherosclerosis. Intriguingly, in animal model of recurrent stroke, rapid neutrophil NETosis was identified as the main source of cell-free DNA after stroke and NET–DNA as the causative agent leading to inflammasome activation (50). It is postulated that DNA-sensing inflammasomes may further cause amplified inflammation in atherosclerotic plaques accounting for recurrent atherosclerotic stroke, and targeting NETs, inflammasome and downstream IL-1β may take effect in reducing residual vascular risk. The notion is strongly supported by data from the CANTOS trial (Canakinumab Anti-Inflammatory Thrombosis Outcome Study) showing that treatment with an IL1β-blocking antibody decreases the risk of a recurrent cardiovascular event in patients with established atherosclerotic disease (51). However, treatment with Canakinumab also comes with heightened risk for infection, a side effect that may not be neglect (51). The challenge now is to find out novel drug target which can achieve the goal of ischemic stroke secondary prevention and with low risk of side effects so that stroke patients can be treated for their disease over decades (33, 50). Comparison with global inhibition of IL1β, the side effect of infection may not be assumed by specific inhibition of upstream component of this pathway, such as NETs and AIM2 inflammasome (33, 50). This notion is based on the understanding that Aim2 senses damage-associated molecular patterns, such as NET-DNA, accumulating in sterile inflammation; its inhibition may add the degree of selectivity toward the cause of the sterile inflammation in comparison with global inhibition of IL1β (33). Furthermore, neutralization of NET-DNA by DNase treatment or inhibition of inflammasome activation reduced the rate of stroke recurrence after experimental stroke (50). It is postulated that treatment target NETs/AIM2 inflammasome pathway may effectively alleviate the pro-inflammatory and pro-thrombotic activity of aggregated NETs, which represents a promising avenue for further clinical development in the precise treat and prevention of LAA stroke in the future.

This research indicated NETs and related inflammatory biomarkers at baseline independently predicted outcome at 3 months and late major vascular events following LAA stroke, suggesting that targeted therapy directed at high-risk patients with elevated baseline inflammation may be beneficial. However, this study had some limitations. Firstly, we focused on LAA stroke in the study and these findings might not be applicable to cardioembolic stroke and other subtype of ischemic stroke. Additionally, this was a single-center study in Chinese population, which might not be generalized to other racial stroke patients. Future multi-center studies with large-sample size are needed to confirm these results. Finally, our study is a prospective clinical observational study aimed at identifying new biomarkers associated with the residual risk of recurrent events in LAA stroke, which revealed NETs and related inflammatory biomarkers at baseline predicted late major vascular events following LAA stroke. To validate the safety and efficacy of targeting NETs and other components of this pathway to reduce residual vascular risk after LAA stroke, future preclinical studies and randomized trials are warranted.

## 5 Conclusions

NETs and related inflammatory biomarkers at baseline predicted outcome at 3 months and late major vascular events following LAA stroke, suggesting that targeted therapy directed at high-risk patients with elevated baseline inflammation may be beneficial. Our results supported a rationale of randomized trials for targeted therapy directed at high-risk patients with elevated baseline inflammation which characterized with high levels of NETs and related inflammatory biomarkers.

## Data Availability

The original contributions presented in the study are included in the article/**Supplementary Material**. Further inquiries can be directed to the corresponding author.
