# Supplementary material for: Associations of NETs with inflammatory risk and clinical predictive value in large artery atherosclerosis stroke: a prospective cohort study
Source: Front Immunol. 2024 Dec 16;15:1488317. doi: 10.3389/fimmu.2024.1488317 (PMC11682974; doi:10.3389/fimmu.2024.1488317)
Supplement: Supplementary file 1 [file DataSheet1.docx]

**Supplement materials**

**Supplement Table 1.** Baseline clinical and procedural characteristics in LAA stroke and Healthy controls

|  | LAA stroke | Healthy controls | | *P* value | |
| --- | --- | --- | --- | --- | --- |
| Age, years | 65.0±10.3 | | 64.4±7.8 | 0.585 | |
| Sex, female | 47（32.4%） | | 53（43.8%） | 0.056 | |
| Smoking | 46（31.7%） | | 14（11.6%） | <0.001 | |
| Hypertension | 109（75.2%） | | 11（9.1%） | <0.001 | |
| Diabetes mellitus Type 2 | 40（27.6%） | | 6 (5.0%） | <0.001 | |
| LDL-C, mmol/L | 2.74 (2.15, 3.17) | | 2.81 (2.07, 3.37) | | 0.467 |
| HDL-C, mmol/L | 1.07 (0.93, 1.25) | | 1.00 (0.72, 1.31) | | 0.090 |
| Triglycerides, mmol/L | 1.46 (1.11, 2.14) | | 1.19 (0.91, 1.92) | | 0.025 |
| Cholesterol, mmol/L | 4.63 (3.99 5.30) | | 4.27 (3.41, 5.21) | | 0.031 |

LDL-C: Low-Density Lipoprotein Cholesterol

HDL-C: High-Density Lipoprotein Cholesterol

**Supplement Table 2.** Correlation between NIHSS scores and NETs and inflammatory

biomarkers levels at three time points in LAA stroke patients.

|  |  | | MPO-DNA | PAD4 | | HMGB1 | C1q | AIM2 |
| --- | --- | --- | --- | --- | --- | --- | --- | --- |
| NIHSS | Correlation Coefficient | 0.076 | | -0.008 | | 0.217 | -0.065 | -0.152 |
|  | *P* value | 0.366 | | 0.925 | | 0.009 | 0.438 | 0.068 |
|  |  | ASC | | Caspase-1 | | IL-1β | IL-6 | IL-8 |
| NIHSS | Correlation Coefficient | 0.012 | | 0.015 | | 0.073 | 0.095 | 0.112 |
|  | *P* value | 0.887 | | 0.854 | | 0.385 | 0.256 | 0.180 |
|  |  |  | | (a) |  | |  |  |

|  |  | | MPO-DNA | PAD4 | | HMGB1 | C1q | AIM2 |
| --- | --- | --- | --- | --- | --- | --- | --- | --- |
| NIHSS | Correlation Coefficient | -0.087 | | 0.107 | | -0.134 | -0.146 | -0.023 |
|  | *P* value | 0.298 | | 0.202 | | 0.107 | 0.081 | 0.785 |
|  |  | ASC | | Caspase-1 | | IL-1β | IL-6 | IL-8 |
| NIHSS | Correlation Coefficient | 0.139 | | -0.002 | | -0.085 | 0.003 | 0.060 |
|  | *P* value | 0.095 | | 0.983 | | 0.311 | 0.971 | 0.477 |
|  |  |  | | (b) |  | |  |  |

|  |  | | MPO-DNA | PAD4 | | HMGB1 | C1q | AIM2 |
| --- | --- | --- | --- | --- | --- | --- | --- | --- |
| NIHSS | Correlation Coefficient | -0.052 | | -0.053 | | -0.184 | -0.108 | 0.020 |
|  | *P* value | 0.584 | | 0.576 | | 0.052 | 0.257 | 0.831 |
|  |  | ASC | | Caspase-1 | | IL-1β | IL-6 | IL-8 |
| NIHSS | Correlation Coefficient | -0.102 | | -0.013 | | 0.061 | -0.116 | 0.108 |
|  | *P* value | 0.283 | | 0.894 | | 0.525 | 0.223 | 0.258 |
|  |  |  | | (c) |  | |  |  |

Table 2a: Correlation analysis between NIHSS and NETs and inflammatory biomarker levels at T1.

Table 2b: Correlation analysis between NIHSS and NETs and inflammatory biomarker levels at T2.

Table 2c: Correlation analysis between NIHSS and NETs and inflammatory biomarker levels at T3.

T1: 24 hours after stroke onset.

T2: 48 hours after stroke onset.

T3: 7 days after stroke onset.

**Supplement Table 3.** NETs and inflammatory biomarkers ROC curve at three times in LAA stroke patients and Healthy controls

| **Factors** |  | **AUC** | **Cut-off** | ***P*** |
| --- | --- | --- | --- | --- |
| MPO-DNA (ng/mL) | 24h | 0.708 | >11.5 | <0.001 |
|  | 48h | 0.678 | >10.45 | <0.001 |
|  | 7d | 0.624 | >11.5 | 0.001 |
| PAD4 (ng/mL) | 24h | 0.632 | >6.78 | <0.001 |
|  | 48h | 0.554 | >8.31 | 0.132 |
|  | 7d | 0. 573 | >8.31 | 0.053 |
| HMGB1 (ng/mL) | 24h | 0.568 | >21.9 | 0.061 |
|  | 48h | 0.669 | >21.9 | <0.001 |
|  | 7d | 0.537 | >21.49 | 0.372 |
| C1q (ug/mL) | 24h | 0.912 | >604.87 | <0.001 |
|  | 48h | 0.883 | >604.87 | <0.001 |
|  | 7d | 0.770 | >564.3 | <0.001 |
| AIM2 (ng/mL) | 24h | 0.931 | >2.6 | <0.001 |
|  | 48h | 0.927 | >2.6 | <0.001 |
|  | 7d | 0.923 | >2.53 | <0.001 |
| ASC (pg/mL) | 24h | 0.661 | >202.52 | <0.001 |
|  | 48h | 0.651 | >202.52 | <0.001 |
|  | 7d | 0.691 | >214.27 | <0.001 |
| Caspase-1 (pg/mL) | 24h | 0.737 | >28.92 | <0.001 |
|  | 48h | 0.716 | >28.92 | <0.001 |
|  | 7d | 0.675 | >25.93 | <0.001 |
| IL-1β (pg/mL) | 24h | 0.702 | >60.91 | <0.001 |
|  | 48h | 0.808 | >59.79 | <0.001 |
|  | 7d | 0.704 | >60.91 | <0.001 |
| IL-6 (pg/mL) | 24h | 0.835 | >7.14 | <0.001 |
|  | 48h | 0.722 | >7.14 | <0.001 |
|  | 7d | 0.651 | >7.14 | <0.001 |
| IL-8 (pg/mL) | 24h | 0.601 | >1736.92 | 0.007 |
|  | 48h | 0.589 | >1736.92 | 0.014 |
|  | 7d | 0.709 | >1294.61 | <0.001 |

**Supplement Table 4.** Logistic regression analysis of inflammatory biomarkers at 48 hours after LAA stroke onset and poor clinical outcomes.

| Variables | Univariate | | |  | Multivariate | | |
| --- | --- | --- | --- | --- | --- | --- | --- |
|  | OR | 95%CI | *P* |  | OR | 95%CI | *P* |
| Age（≥60） | 2.65 | 1.18-5.64 | 0.018 |  | 2.91 | 1.25-6.74 | 0.012 |
| Sex（female） | 2.35 | 1.13-4.90 | 0.022 |  | - | - | - |
| Hypertension | 1.07 | 0.47-2.42 | 0.862 |  | - | - | - |
| T2DM | 3.04 | 1.41-6.51 | 0.004 |  | 3.03 | 1.49-7.29 | 0.003 |
| Smoke | 0.67 | 0.31-1.46 | 0.322 |  | - | - | - |
| BMI (≥28kg/m^2^) | 1.22 | 0.38-3.86 | 0.736 |  | - | - | - |
| LDL-C (≥3.4mmol/L) | 1.35 | 0.55-3.21 | 0.512 |  | - | - | - |
| HDL-C（≤1.16mmol/L) | 0.93 | 0.45-1.93 | 0.851 |  | - | - | - |
| Triglycerides (≥1.7mmol/L) | 0.86 | 0.42-1.77 | 0.693 |  | - | - | - |
| Cholesterol (≥5.2mmol/L) | 1.11 | 0.51-2.38 | 0.790 |  | - | - | - |
| Hcy (≥15mmol/L) | 1.53 | 0.69- 3.36 | 0.288 |  | - | - | - |
| MPO-DNA (≥10.45ng/mL) | 0.50 | 0.21-1.19 | 0.119 |  | - | - | - |
| PAD4 (≥8.31ng/mL) | 1.03 | 0.50-2.11 | 0.932 |  | - | - | - |
| HMGB1 (≥21.9ng/mL) | 1.48 | 0.73-3.00 | 0.268 |  | - | - | - |
| C1q (≥604.87ug/mL) | 1.07 | 0.47-2.42 | 0.862 |  | - | - | - |
| AIM2 (≥2.6ng/mL) | 0.97 | 0.40-2.36 | 0.958 |  | - | - | - |
| ASC (≥202.52pg/mL) | 0.64 | 0.31-1.32 | 0.237 |  | - | - | - |
| Caspase-1 (≥28.92pg/mL) | 1.28 | 0.63-2.62 | 0.484 |  | - | - | - |
| IL-1β (≥59.79pg/mL) | 0.81 | 0.39-1.63 | 0.552 |  | - | - | - |
| IL-6 (≥7.14pg/mL) | 1.03 | 0.50-2.11 | 0.932 |  | - | - | - |
| IL-8 (≥1736.92pg/mL) | 0.47 | 0.22-1.03 | 0.061 |  | - | - | - |

OR: Odds Ratio, CI: Confidence Interval

**Supplement Table 5.** Logistic regression analysis of inflammatory biomarkers at 7 days after LAA stroke onset and poor clinical outcomes.

| Variables | Univariate | | |  | Multivariate | | |
| --- | --- | --- | --- | --- | --- | --- | --- |
|  | OR | 95%CI | *P* |  | OR | 95%CI | *P* |
| Age（≥60） | 2.65 | 1.18-5.64 | 0.018 |  | 2.94 | 1.00-8.62 | 0.049 |
| Sex（female） | 2.35 | 1.13-4.90 | 0.022 |  | 2.77 | 1.07-7.13 | 0.035 |
| Hypertension | 1.07 | 0.47-2.42 | 0.862 |  | - | - | - |
| T2DM | 3.04 | 1.41-6.51 | 0.004 |  | 4.78 | 1.80-12.6 | 0.002 |
| Smoke | 0.67 | 0.31-1.46 | 0.322 |  | 2.94 | 1.00-8.62 | 0.049 |
| BMI (≥28kg/m^2^) | 1.22 | 0.38-3.86 | 0.736 |  | - | - | - |
| LDL-C (≥3.4mmol/L) | 1.35 | 0.55-3.21 | 0.512 |  | - | - | - |
| HDL-C（≤1.16mmol/L) | 0.93 | 0.45-1.93 | 0.851 |  | - | - | - |
| Triglycerides (≥1.7mmol/L) | 0.86 | 0.42-1.77 | 0.693 |  | - | - | - |
| Cholesterol (≥5.2mmol/L) | 1.11 | 0.51-2.38 | 0.790 |  | - | - | - |
| Hcy (≥15mmol/L) | 1.53 | 0.69- 3.36 | 0.288 |  | - | - | - |
| MPO-DNA (≥11.5ng/mL) | 0.57 | 0.23-1.40 | 0.908 |  | - | - | - |
| PAD4 (≥8.31ng/mL) | 1.32 | 0.64-2.74 | 0.226 |  | - | - | - |
| HMGB1 (≥21.49ng/mL) | 0.48 | 0.19-1.22 | 0.127 |  | - | - | - |
| C1q (≥564.3ug/mL) | 0.77 | 0.32-1.84 | 0.567 |  | - | - | - |
| AIM2 (≥2.53ng/mL) | 0.85 | 0.31-2.33 | 0.766 |  | - | - | - |
| ASC (≥214.27pg/mL) | 1.12 | 0.54-2.31 | 0.748 |  | - | - | - |
| Caspase-1 (≥25.93pg/mL) | 1.67 | 0.71-3.88 | 0.233 |  | - | - | - |
| IL-1β (≥60.91pg/mL) | 1.51 | 0.65-3.53 | 0.335 |  | - | - | - |
| IL-6 (≥7.14pg/mL) | 0.82 | 0.32-2.11 | 0.695 |  | - | - | - |
| IL-8 (≥1294.61pg/mL) | 2.28 | 0.96-5.36 | 0.059 |  | - | - | - |

OR: Odds Ratio, CI: Confidence Interval

Figure 1. Correlation analysis with the level of NETs and inflammatory biomarkers in the acute phase of LAA stroke.


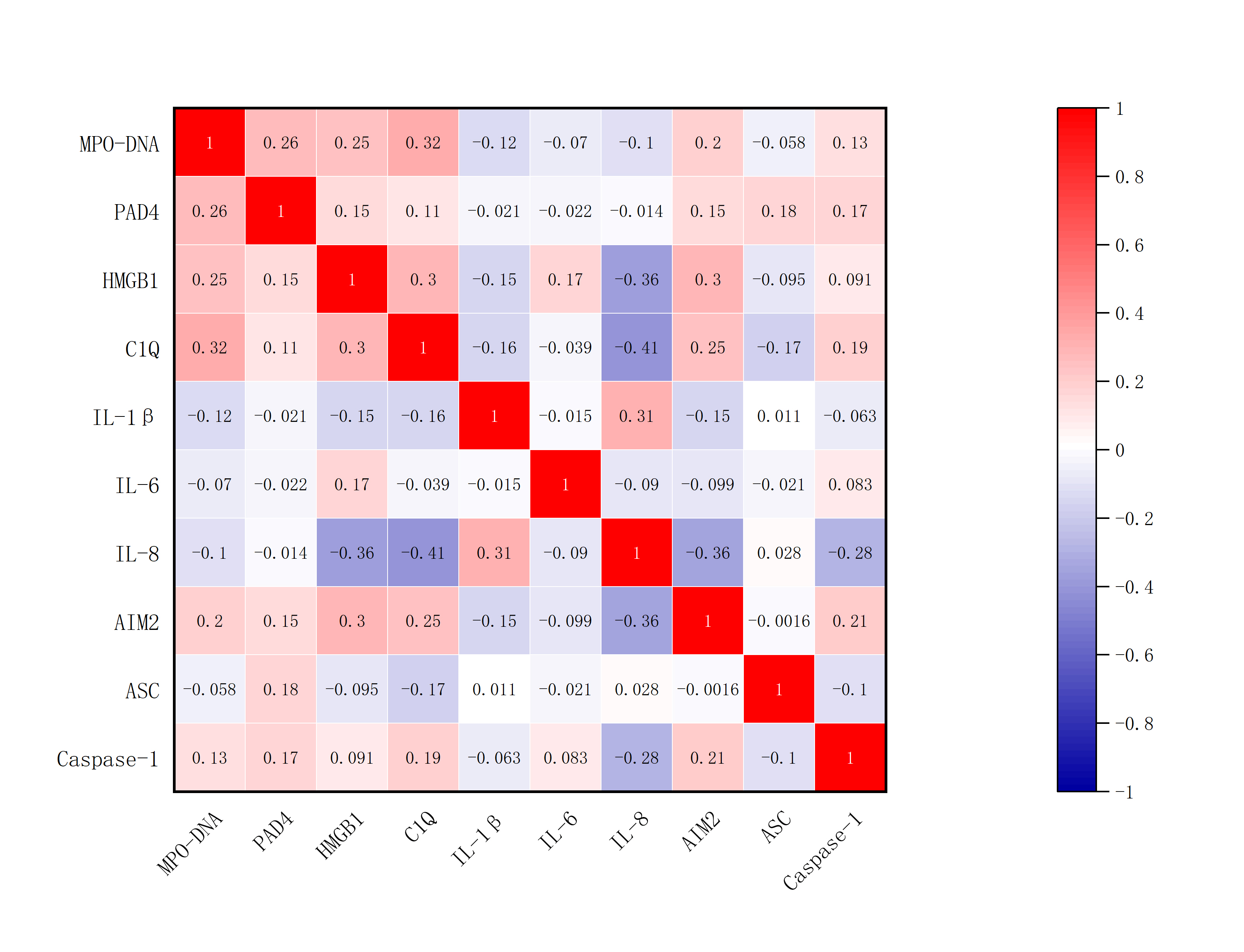


(a)


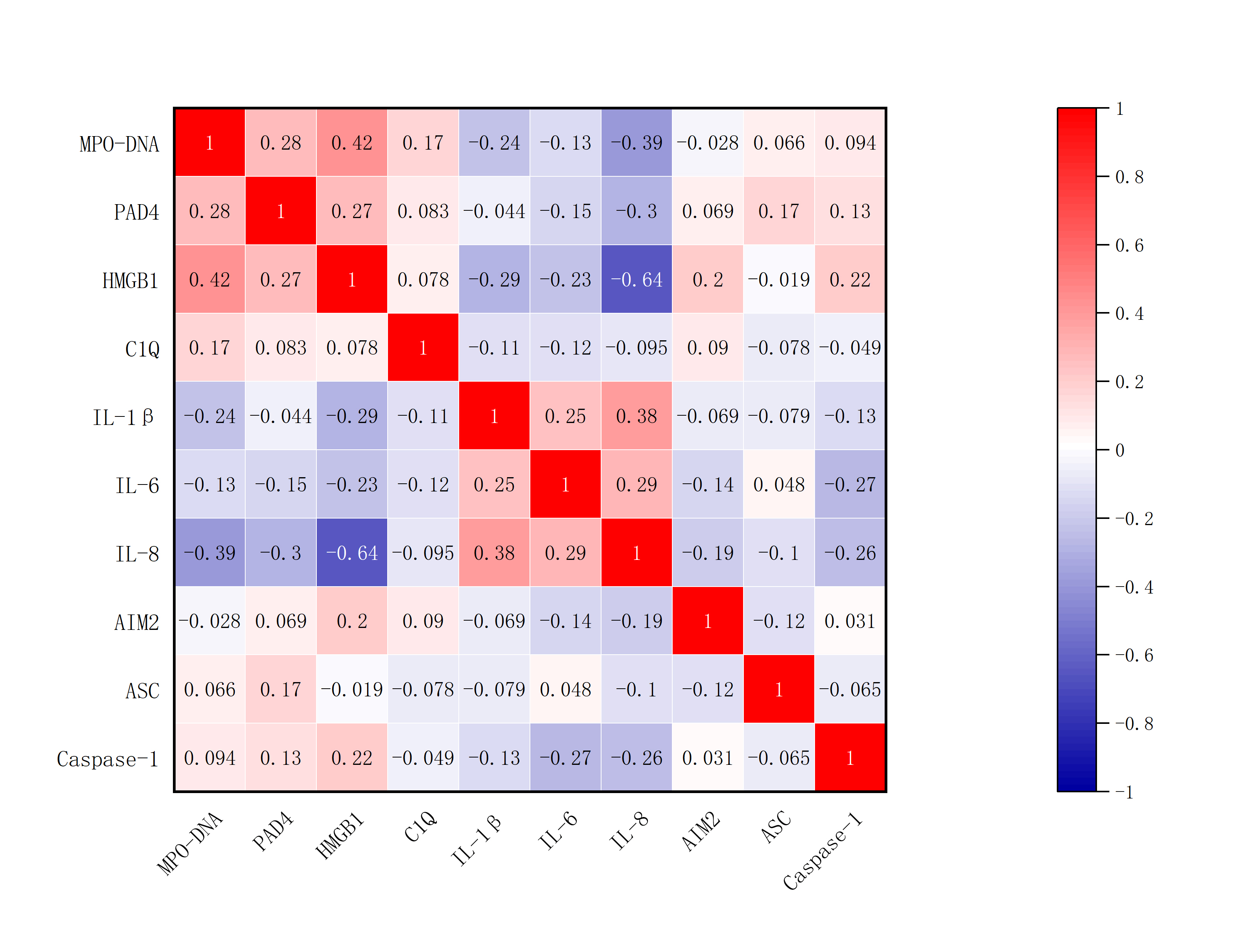


(b)


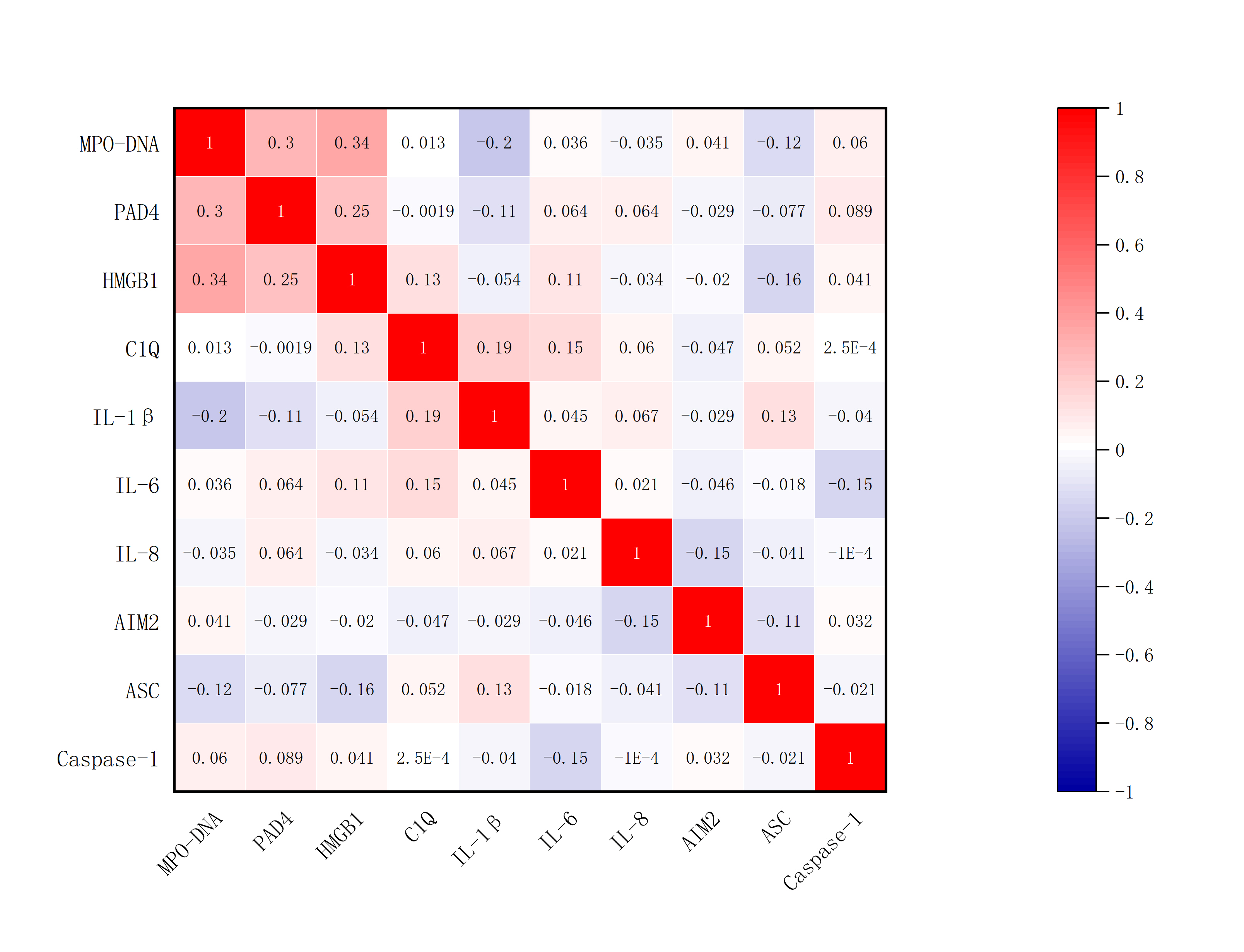


(c)

Figure 1a: Correlation analysis between NETs and inflammatory Biomarkers at T1.

Figure 1b: Correlation analysis between NETs and inflammatory Biomarkers at T2.

Figure 1c: Correlation analysis between NETs and inflammatory Biomarkers at T3.

T1: 24 hours after stroke onset.

T2: 48 hours after stroke onset.

T3: 7 days after stroke onset.
